# Capturing enzymes in motion

**DOI:** 10.7554/eLife.108727

**Published:** 2025-09-24

**Authors:** Álvaro de la Gándara, Agnieszka A Kendrick

**Affiliations:** 1 Salk Institute for Biological Studies La Jolla United States

**Keywords:** angioten-I converting enzyme, hypertension, amyloid peptide, cryo-EM, all-atom MD simulations, enzyme dynamics, cryo-EM heterogeneity analysis, Human

## Abstract

A combination of cryogenic electron microscopy and molecular simulations has been used to study the structure and dynamics of an enzyme called ACE.

**Related research article** Mancl JM, Wu X, Zhao M, Tang WJ. 2025. Dimerization and dynamics of angiotensin-I converting enzyme revealed by Cryo-EM and MD simulations. *eLife* **14**:RP106044. doi: 10.7554/eLife.106044.

Every second of your life, an invisible conversation unfolds within your body that determines how your heart beats, blood flows and brain functions. The ‘words’ in this conversation are bioactive peptides, tiny molecules that act as cellular messengers. These words are edited by enzymes that refine and modify the message, ensuring the dialogue is clear and on point. But when the editors fail, the conversation breaks down, disrupting the body’s balance and driving disease.

An important enzyme for modifying a wide range of peptides – including those central to cardiovascular and renal health – is the angiotensin-I converting enzyme or ACE (Herman et al., 2023). ACE is also linked to Alzheimer’s disease, where its role is more complicated. While ACE can degrade amyloid β, whose build-up in the brain is related to the disease, inhibiting the activity of ACE can benefit some Alzheimer’s disease patients (Gouveia et al., 2022). Thus, a greater understanding of ACE could lead to advancements in therapies for renal, cardiovascular and neurodegenerative diseases.

At the molecular level, ACE is a membrane-anchored protein with two catalytic domains, ACE-N and ACE-C, that can form homodimers. ACE can be released into the extracellular region as soluble ACE, where it also exists as a homodimer (Figure 1A; Wei et al., 1991; Kost et al., 2000). Structural studies indicate that each catalytic domain prefers a different peptide target or substrate, and that they can influence each other’s activity, suggesting a dynamic behaviour (Acharya et al., 2024).

**Figure 1.**
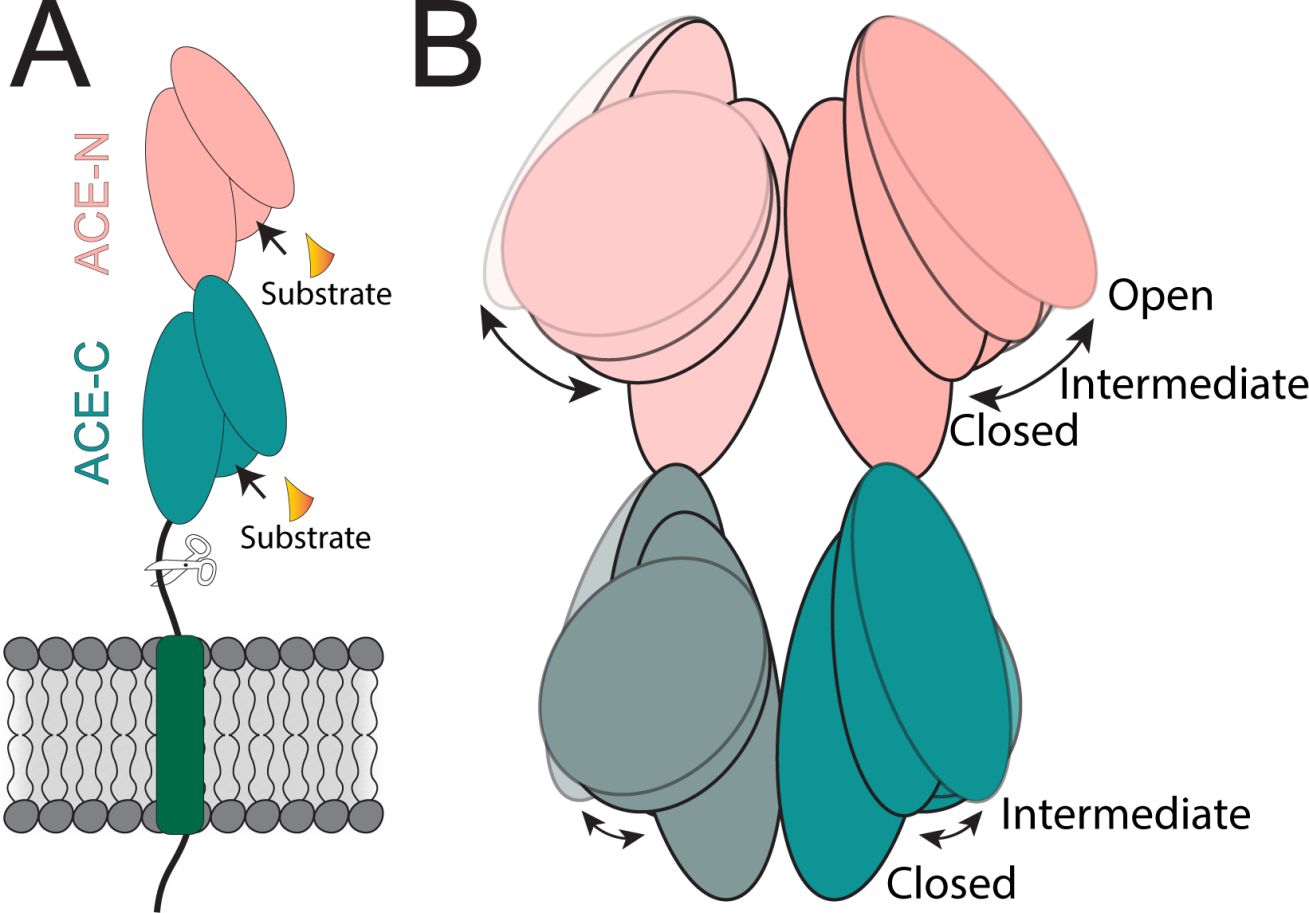
The soluble ACE dimer exhibits dynamic behavior in its catalytic pocket. (**A**) The angiotensin-I converting enzyme (ACE) is a transmembrane protein composed of two homologous catalytic domains, ACE-N (peach) and ACE-C (green). Each domain contains three subdomains that, together, form a catalytic pocket for peptide binding. Soluble ACE is generated by proteolytic cleavage (indicated by scissors) and is released into the extracellular space. (**B**) In solution, ACE forms a homodimer that displays dynamic behavior, with ACE-N showing greater flexibility (being observed in three states: closed, intermediate and open) compared to ACE-C (closed and intermediate states). Among the three subdomains, two exhibit the highest mobility, driving the opening and closing of the catalytic pocket.

However, how ACE accommodates its full range of substrates – and how it functions as a dimer – remains unclear. Now, in eLife, Jordan Mancl, Xiaoyang Wu, Minglei Zhao, and Wei-Jen Tang of the University of Chicago report the results of a cryogenic electron microscopy (cryo-EM) study that sheds new light on these questions (Mancl et al., 2025).

Cryo-EM is a powerful structural tool for analyzing dynamic protein behavior. Preserving proteins in a thin and rapidly frozen aqueous layer can maintain their structure and enable visualization of multiple conformations. Coupled with computational approaches that enhance the detection of rare states, cryo-EM can be an effective way to capture the dynamic behavior of enzymes. Previous cryo-EM studies of soluble ACE suggested a flexible structure but lacked molecular details, likely due to protein damage during sample preparation (Lubbe et al., 2022).

Mancl et al. demonstrated that soluble ACE adopts different conformations in its catalytic domains, providing potential insights into how it binds various peptide substrates. Each catalytic domain consists of three subdomains, whose relative positions regulate access to the catalytic “pocket” where the substrate binds and is modified by ACE (Figure 1A). Based on interdomain distances within this pocket, the researchers identified three domain-specific conformational states: open, intermediate and closed. ACE-N was more flexible than ACE-C, being observed in all three states, while ACE-C was consistently either in the intermediate or closed state (Figure 1B). Both domains use hydrophobic interactions to stabilize the closed state. However, the reduced hydrophobic interface in ACE-N likely favors more open states.

Mancl et al. also explored the dimerization interface in soluble ACE. They showed that although ACE-N and ACE-C share the same overall fold, distinct residues mediate the formation of the homodimer, with ACE-N forming more extensive contacts. N-terminal glycosylation further reinforces ACE-N dimerization, whereas fewer interactions and a lesser contribution from glycosylation in ACE-C could explain its reduced stability and tendency to denature during structural studies.

To further probe soluble ACE dynamics, Mancl et al. mapped the motions of the catalytic domains by combining multiple cryo-EM heterogeneity analysis approaches and molecular dynamics simulations. They found that each dimer moves independently, with dynamics dominated by opening and closing of the catalytic pockets, rather than large displacement between the domains (Figure 1B). Although the dimerization subdomains remain highly stable, Mancl et al. suggest that the connection between them could govern allosteric communication between ACE-N and ACE-C. Together, these results suggest that dimerization stabilizes the enzyme, while subdomain mobility governs substrate binding (Lubbe et al., 2022).

These findings support a model in which ACE uses an open–close substrate capture mechanism, and the distinct conformational states of ACE-N and ACE-C likely underlie their substrate specificities. Greater flexibility in ACE-N could explain why it favors larger peptides such as amyloid β (Larmuth et al., 2016). These mechanistic insights point to refined therapeutic strategies, where rather than fully inhibiting ACE, drugs could modulate domain dynamics to influence substrate specificity.

The work of Mancl et al. elegantly combines structural biology with computational approaches to sketch a coherent model of protein dynamics, and to propose a new rationale for therapeutic targeting. While studies of soluble ACE bound to its substrates will be needed to fully understand its function, Mancl et al. establish a framework for others to use when studying ACE and other enzyme dynamics.
